# Early detection of anthropogenic climate change signals in the ocean interior

**DOI:** 10.1038/s41598-023-30159-0

**Published:** 2023-02-21

**Authors:** Jerry F. Tjiputra, Jean Negrel, Are Olsen

**Affiliations:** 1 NORCE Norwegian Research Centre, Bjerknes Centre for Climate Research, Bergen, Norway; 2 Geophysical Institute, University of Bergen, Bjerknes Centre for Climate Research, Bergen, Norway

**Keywords:** Climate and Earth system modelling, Carbon cycle

## Abstract

Robust detection of anthropogenic climate change is crucial to: (i) improve our understanding of Earth system responses to external forcing, (ii) reduce uncertainty in future climate projections, and (iii) develop efficient mitigation and adaptation plans. Here, we use Earth system model projections to establish the detection timescales of anthropogenic signals in the global ocean through analyzing temperature, salinity, oxygen, and pH evolution from surface to 2000 m depths. For most variables, anthropogenic changes emerge earlier in the interior ocean than at the surface, due to the lower background variability at depth. Acidification is detectable earliest, followed by warming and oxygen changes in the subsurface tropical Atlantic. Temperature and salinity changes in the subsurface tropical and subtropical North Atlantic are shown to be early indicators for a slowdown of the Atlantic Meridional Overturning Circulation. Even under mitigated scenarios, inner ocean anthropogenic signals are projected to emerge within the next few decades. This is because they originate from existing surface changes that are now propagating into the interior. In addition to the tropical Atlantic, our study calls for establishment of long-term interior monitoring systems in the Southern Ocean and North Atlantic in order to elucidate how spatially heterogeneous anthropogenic signals propagate into the interior and impact marine ecosystems and biogeochemistry.

## Introduction

Anthropogenic activities over the past two centuries have led to rapid climate change that is unprecedented over the last two millenniums. In addition, the current warming rate exceeds previous warming as reconstructed from the last 100,000 years^1^. In the ocean, substantial changes have been observed and marine ecosystem impacts from ongoing circulation changes, warming, deoxygenation, and acidification appear unavoidable^2,3^. Isolating anthropogenically-forced signals in the presence of background natural variability, whose magnitude is often non-negligible and spatially complex, is challenging^4^. Due to the ocean’s role in buffering ongoing climate change and in providing essential services to humankind, improved understanding and monitoring of anthropogenic climate change in marine systems are important research priorities.

An optimal long-term monitoring network should provide observations in key regions that allow us to: (i) reaffirm our understanding of Earth system response to external forcing, (ii) improve our predictive capability and (iii) assist ecosystem management and mitigation strategies^5^. While remotely-sensed observations provide invaluable large-scale measurements, they are limited to surface properties. Interior ocean properties are traditionally measured at dedicated research cruises, requiring increasingly high resources. Over the past few decades, autonomous observing platforms such as ARGO profiling floats and gliders have been deployed successfully with a growing number of sensors^6^. The current ARGO array comprises approximately 4000 profiling floats, but remains challenging to sustain due to financial and human constraints^7^. Knowledge on when and where anthropogenic climate change signals will become detectable will help us to guide and prioritize future observational campaigns.

The timing and location of anthropogenic signal emergence for key environmental drivers, such as temperature, oxygen, and pH, are used to assess marine ecosystem vulnerability to exposure beyond natural variability^8^. Rapid emergence gives organisms less time to adapt, implying a high vulnerability. Furthermore, emergence of multiple environmental stressors leads to higher vulnerability than a single stressor since marine biotas would have to concurrently adjust to multiple levels of disruptions. Understanding how these aggregated changes may propagate is vital to identify regions where the overall ecosystem structure, functioning and services that people and societies depend on, will likely be perturbed^9^.

Recent studies have investigated the time of emergence of climate change in the surface ocean^10,11^. Far less have been done for the ocean below the surface^12,13^. In the mesopelagic, climate change is expected to threaten ocean biodiversity even under an aggressive mitigation pathway^14^. Changes in interior thermal range could expose pelagic organisms to novel environmental conditions, further increasing their vulnerability^15^. This highlights the urgent need to identify which and where subsurface environmental drivers will be most affected by climate change and importantly, the time scales at which they will become detectable.

Here, we analyse projections of four Essential Climate Variables as defined by the Global Climate Observing System^16^: temperature, salinity, oxygen, and pH (an indicator for ocean acidification) at different depth levels, and determine the time when the anthropogenically-forced signals will depart from the background internal variability. Our primary objective is to identify consistent environmental changes across an ensemble of CMIP6 (Coupled Model Intercomparison Project phase 6) Earth system models, and assess the impact of climate mitigation. Based on our findings, we assess the current observing network and provide recommendations for optimal future monitoring system networks.

## Results and discussion

We first present and discuss the multi-model mean time of departure (ToD; see “Methods”) for each variable as projected under the high-$\text {CO}_2$ SSP5-8.5 scenario. Throughout the paper, we also use ToD to indicate when anthropogenically-forced signals will become detectable. The general physical and biogeochemical drivers of the spatial ToD patterns are articulated and compared with earlier studies. Next, we present the impact of climate mitigation on the ToD patterns, using the SSP2-4.5 scenario. Lastly, the departure/detectability time scale of multiple drivers in different regions and depth levels are presented.

### Temperature

**Figure 1:**
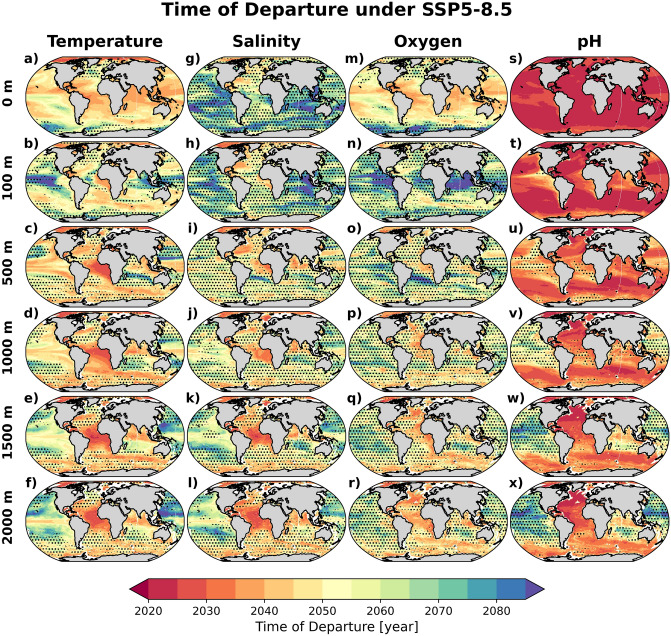
Time of Departure (ToD) maps for SSP5-8.5. Multi-model mean of ToDs for (**a**–**f**) temperature, (**g**–**l**) salinity, (**m**–**r**) oxygen, and (**s**–**x**) pH for surface, 100 m, 500 m, 1000 m, 1500 m and 2000 m depths. Values are estimated from up to 17 Earth system model projections under the SSP5-8.5 future scenario (see also Table 1). The stippling marks areas where the inter-model standard deviation is above 20 years. Maps were generated with Python 3.9.16 using Matplotlib v3.6.2 (https://matplotlib.org/) and Cartopy v0.21.1 (https://scitools.org.uk/cartopy/).

The ToD for temperature at different depth levels under the SSP5-8.5 scenario are shown in Fig. 1a–f. The model projections indicate that anthropogenically-induced warming will be detectable in nearly all parts of the global ocean before the end of the $21{{\text {st}}}$ century, but with large variations in space.

At the ocean surface, ToDs as early as 2030 are projected in the Arctic followed by the tropical oceans, excluding the central parts of the equatorial Pacific and waters off northwestern Africa. In most of the surface ocean, the warming ToDs are robust (i.e. good agreement between models), with a few exceptions in the subpolar North Atlantic and the polar Southern Ocean, where the ToDs are later than 2070. At 100 m, the model projections indicate that early departure regions are limited to the Arctic, the eastern equatorial Atlantic, and parts of the Southern Ocean, while detectability is strongly delayed in the tropical Pacific (Fig. 1b). The latter feature is a consequence of both the simulated low warming rate and the large background variability ($\sigma >1 \;\;^\circ \text {C}$), associated with the El Niño Southern Oscillation^17^ (Figs. 1b, S1b, and S2b). Similarly, the models also simulate late ToD in the top 100 m of the polar Southern Ocean where the warming signal is relatively weak and the natural variability is non-negligible.

**Figure 2:**
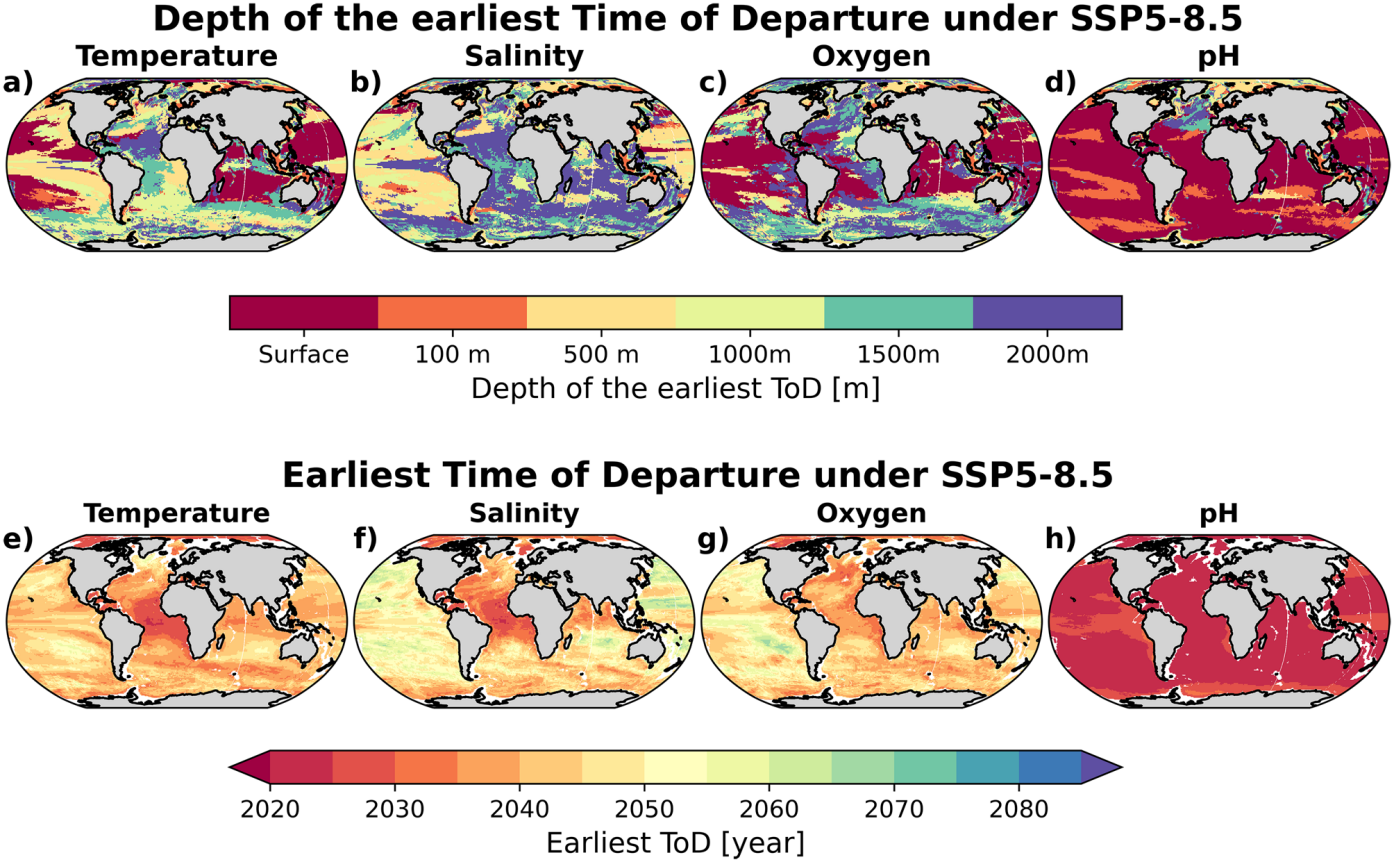
Depth of the earliest departure estimated from the multi-model mean of ToD for (**a**) temperature, (**b**) salinity, (**c**) oxygen, and (**d**) pH. And earliest departure, considering all depths in (**a**–**d**), in the multi-model mean of ToD for (**e**) temperature, (**f**) salinity, (**g**) oxygen, and (**h**) pH. Values are estimated from Earth system model projections under the SSP5-8.5 future scenario. Maps were generated with Python 3.9.16 using Matplotlib v3.6.2 (https://matplotlib.org/) and Cartopy v0.21.1 (https://scitools.org.uk/cartopy/).

The spatial patterns of ToDs at 500–2000 m depths are relatively similar (Fig. 1c–f), with robust early warming, ToD < 2030, in the equatorial Atlantic upwelling and the Arctic regions. In the Southern Ocean, the tropical Pacific, and most of the Atlantic, projected warming signals are detectable earlier at these depths than in the surface layers (Fig. 2). Outside these regions, the ToDs are generally later than 2050 and the model spread increases. For instance, in the subtropical Indian and Pacific Oceans, the ToDs between 500 and 2000 m occur towards the end of the $21{{\text {st}}}$ century, which is considerably later than at the surface. In the subpolar and polar Southern Ocean, early ToD at 500 m and deeper can be linked to the ventilation and formation of Antarctic Intermediate and Mode Water, which efficiently transport excess heat from the atmosphere to depth^18,19^. In the North Pacific, where some of the oldest water masses reside, the ToD increases with depth.

The large-scale pattern of ToD in the upper 500 m can broadly be explained by the amplitude of background variability (defined as $\pm 2$ interannual standard deviation over the preindustrial period; Fig. S1) rather than the actual warming strength (Fig. S2). For instance, despite surface warming exceeding $3 \; ^\circ \text {C}$ by the end of the $21{{\text {st}}}$ century in much of the northern hemisphere, the ToD is 2040 or later. In the subpolar North Atlantic, the large variability associated with the North Atlantic Oscillation and subpolar gyre circulation^20,21^ mask the warming signal. In the tropical Indian and Pacific Oceans, the late ToDs at 100 m also coincide with the simulated large background variability. In contrast, at 500 m, the lowest temperature thresholds are located in the tropical oceans, which also have the earliest ToDs (Figs. 1c and 2a). At 1000 m and below, anthropogenic warming weaken, except in the well ventilated North Atlantic^13^. Nevertheless, earliest ToDs are robustly projected in the tropical Atlantic due to the low background variability (Fig. 1d–f).

Our pattern of surface warming ToD is very consistent with earlier studies based on CMIP5 ESMs^10,11^, but with slightly lower values. Our earlier departure timescale can be explained by the difference in the methods used. As pointed out^10^, the timing of signal emergence is sensitive to the applied methods. We have used a 10-years smoothed time-series and standard deviation of the inter-annual variations in our approach, compared to a 30-years interval trend^10^ or the standard deviation of annual extrema^11^ used in earlier studies.

Taking into account the estimated temperature ToD at all six depth levels, Fig. 2a,e summarizes that some of the earliest warming signals would be detectable below the surface, i.e., at 500 m in the equatorial Atlantic (2030s) and at 1000 m in the circumpolar Southern Ocean ( 2040s). In the surface ocean outside the Arctic, warming will be detectable earliest in the tropical Indian and western Pacific ( 2040s). Our findings of warming-induced early ToD below the surface layer is consistent with a recent study that indicates early departure of thermal ranges beyond natural variability in many parts of the interior ocean^15^.

**Figure 3:**
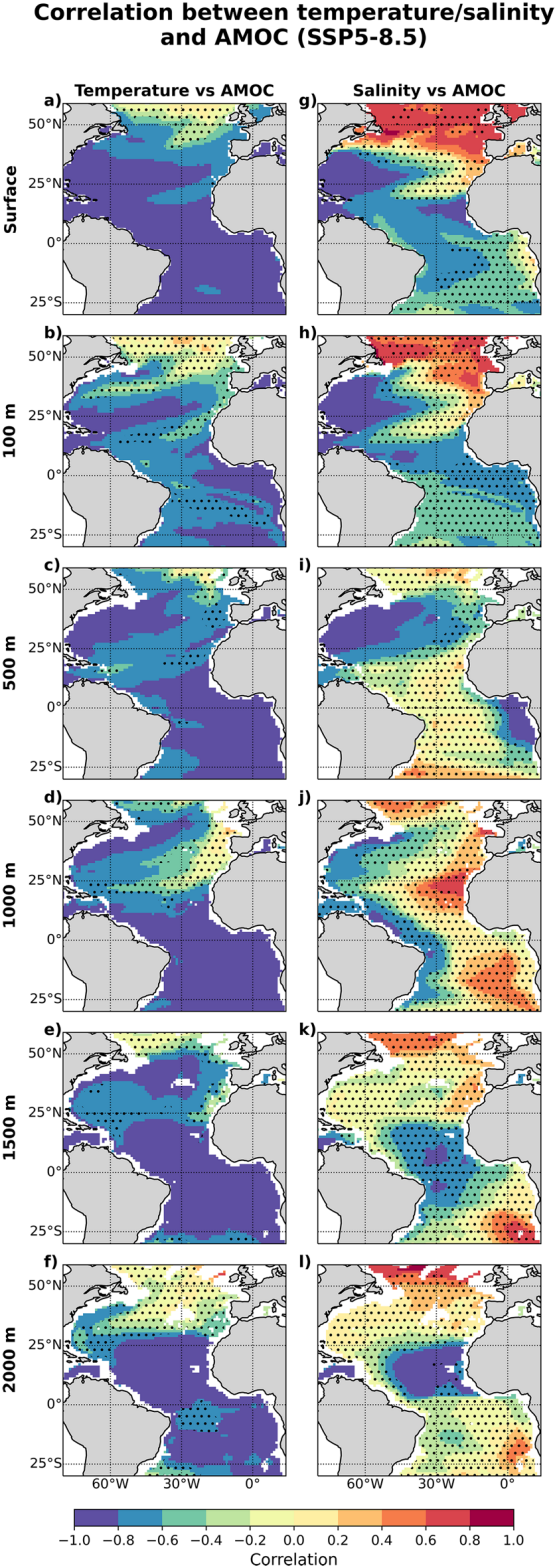
Correlation between trends in temperature/salinity and AMOC strength at 26.5$^\circ$N for the period 2015 to 2100 under the SSP5-8.5 scenario, averaged across all models, at (**a**,**g**) ocean surface, (**b**,**h**) 100 m, (**c**,**i**) 500 m, (**d**,**j**) 1000 m, (**e**,**k**) 1500 m, and (**f**,**l**) 2000 m. The stippling marks represent areas where the inter-model standard deviation is above 0.3. Maps were generated with Python 3.9.16 using Matplotlib v3.6.2 (https://matplotlib.org/) and Cartopy v0.21.1 (https://scitools.org.uk/cartopy/).

**Figure 4:**
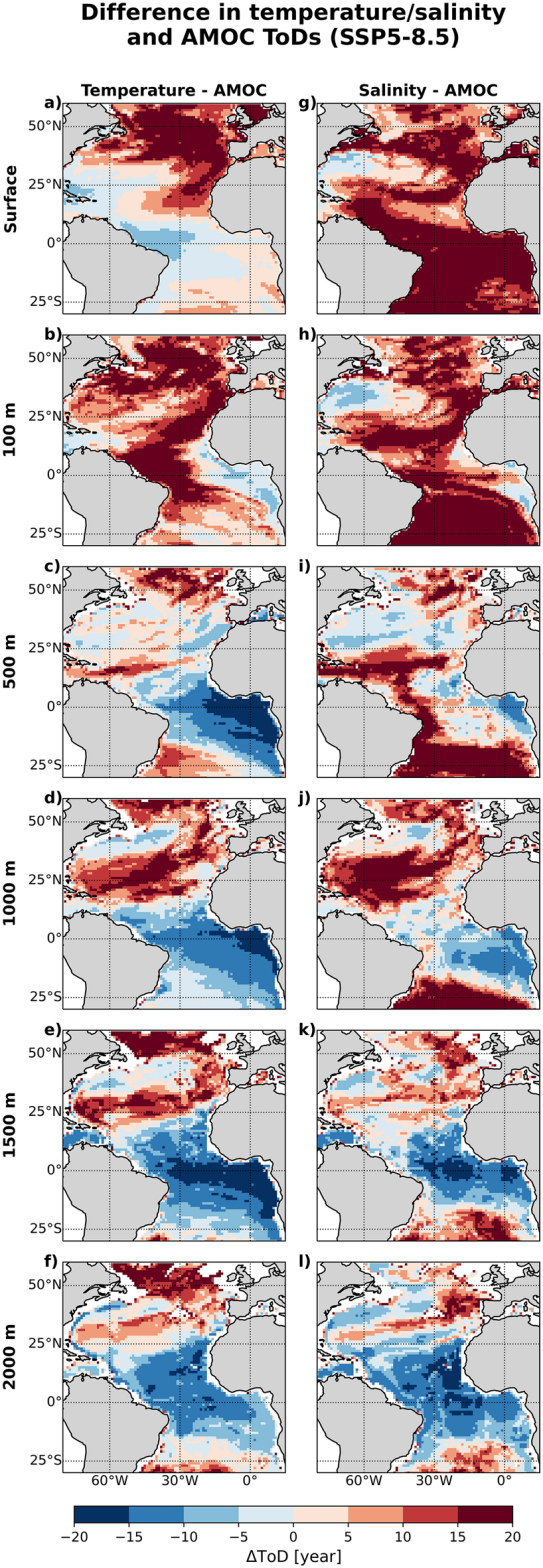
Multi-model mean difference between temperature/salinity and AMOC ToDs under SSP5-8.5 scenario at (**a**,**g**) ocean surface, (**b**,**h**) 100 m, (**c**,**i**) 500 m, (**d**,**j**), 1000 m depths, (**e**,**k**) 1500 m, and (**f**,**l**) 2000 m. Red denotes areas where temperature/salinity changes lag the AMOC signal changes while blue denotes the opposite. Maps were generated with Python 3.9.16 using Matplotlib v3.6.2 (https://matplotlib.org/) and Cartopy v0.21.1 (https://scitools.org.uk/cartopy/).

We also investigate if the early ToDs in the Gulf Stream and tropical Atlantic are linked to the projected decline in Atlantic Meridional Overturning Circulation (AMOC) strength^22^. Figure 3a–f shows that, in the tropics at 500 m or deeper, the correlation between temperature and AMOC is significant ($r<=-0.8$) and consistent across the models. This suggests that the warming signal could partly be driven by upwelling strength, which is connected to the AMOC^23^. Along the Gulf Stream extension region, where early ToDs are simulated, the correlation is also similarly significant between 500 m and 1000 m, suggesting that the AMOC decline could partly contribute to the simulated warming (Fig. S2c,d) through the reduction of northward heat transport or weaker upwelling. Comparing the temperature and AMOC ToDs (see also Supplementary Fig. S3a), Fig. 4a–f shows that in the eastern tropical Atlantic, temperature ToD between 500 and 1500 m lead the AMOC ToD by appoximately 20 years, suggesting that the temperature here could provide an early indicator for future AMOC change.

### Salinity

Projected salinity changes in the water column mostly reflect perturbed circulation patterns and watermass redistribution, whereas in the surface, changes in freshwater loads also play a role^24^. Under the SSP5-8.5 scenario, robust surface salinification occurs in the subtropical and tropical Atlantic and in the southeast subtropical Pacific (Fig. S2g), consistent with the current observed trends^25^. Surface freshening is simulated in the subpolar North Atlantic, the North Pacific, and the western tropical Pacific. These salinity changes, which extend to 100 m depth (Fig. S2h) likely originates from the long-term evaporation and precipitation trends induced by global warming^26,27^. In the Arctic, surface freshening is consistently simulated across the models and is due to sea-ice melting and increased runoff from land^28^.

The stippling patterns in Fig. 1g,h indicate a large inter-model uncertainty in ToDs, despite pronounced salinification and freshening signals in the upper ocean, with a few exceptions, e.g., in the western subtropical North Atlantic and the Arctic. In the former region, a steady salinity increase has been observed in recent decades^25^ and is projected to continue and surpass the relatively weak internal variability (Fig. S1g,h) already in the 2040s. In the Arctic, long-term freshening is projected to be detectable before 2050, from surface to 2000 m depth (Fig. 1g–l), despite the large background variability. In contrast, while the multi-model mean projects that the largest surface freshening will take place in the subpolar North Atlantic (Fig. S2g), this will only be detectable in the second half of $21{{\text {st}}}$ century (with large inter-model spread) due to the large background variability (Fig. S1g).

In parts of the Pacific, south Indian, and most of the Southern Ocean, the ToDs are delayed to the end of the century, with a large inter-model spread. Similar to temperature, for most parts of the global ocean, the earliest detectable salinity changes occur below 100 m depth (Fig. 2b), especially in the tropical Atlantic and along the Gulf Stream (Fig. 2f). In addition, along the sea-ice edges in both hemispheres, where future sea-ice melting could considerably alter watermass salinity prior to being subducted during deepwater formation process, subsurface salinity changes are detectable early (Fig. 1j–l).

The early ToD in the western subtropical North Atlantic is of particular interest. This extends from surface down to 500 m and is the result of a robust salinity increase projected across the models (Figs. 1g–i and S2g–i). This salinity increase is attributed to a weakening of AMOC strength under the SSP5-8.5 scenario^22^, which reduces the poleward transport of saline tropical watermasses. Figure 3g–l shows the negative correlation between AMOC strength and salinity in the western subtropical North Atlantic, and this is significant ($r<=-0.8$) down to 500 m, implying that salinity changes here could also be an indicator for long-term AMOC trends. Similar to temperature, the salinity-AMOC correlation at 2000 m in the northeastern tropical Atlantic is also significant. The difference between salinity and AMOC ToDs (see also Supplementary Fig. S3b), averaged across all models (Fig. 4g–l), indeed shows that salinity changes in the western subtropical North Atlantic (between 100 and 500 m depths) and eastern tropical Atlantic (1500 m and 2000 m) potentially are robust early indicators of AMOC change, providing approximately 10-20 years lead time (i.e. with respect to the emergence of AMOC signal).

### Oxygen

The surface oxygen ToD patterns show a remarkable resemblance with surface temperature ToD. This is expected since the surface oxygen saturation concentration is strongly regulated by temperature. The earliest ToD occur just before the mid $21{{\text {st}}}$ century, in the tropical Atlantic and Indian Oceans and the western subtropical domains (Fig. 1m). In these regions, the oxygen interannual variability is low (Fig. S1m) and the inter-model agreement in ToD is robust and mostly earlier than at depth (Figs. 1m and 2c). In the tropical Pacific, relatively large ENSO-driven oxygen variability delays the surface oxygen ToD. Delayed and more uncertain ToD is also projected in the high latitude regions. Deoxygenation is projected to occur in all parts of the surface ocean (except for the Weddell Sea) by the end of the $21{{\text {st}}}$ century (Fig. S2m), because of the lower solubility of oxygen in the warmer surface ocean.

In contrast to the surface, the ToD at 100 m depth in the tropical oceans are further delayed and only occur toward the end of the century. This result is particularly robust in the Indian and eastern tropical Pacific Oceans (Fig. 1n), where the relatively large interannual variability masks out the projected weak oxygen changes (Figs. S1n and S2n). The relatively large oxygen interannual variability at the base of the euphotic zone is consistent with the large variability of low latitude primary production, which regulates the oxygen consumption below this level^29^. The projected weak change can be attributed to the compensation between a reduced apparent oxygen utilization (AOU) due to the projected productivity decline and reduced oxygen solubility due to warmer temperature^3,12^.

At 500 m and deeper, the oxygen ToD patterns are similar across all layers. Most projected changes will be detectable by the middle of the century (Fig. 1o–r), except in the tropical and subtropical Pacific and subtropical South Atlantic (at 500 m). Robust early departure (2040s) is projected in the eastern tropical Atlantic at 500 m (Fig. 1o), where models simulate oxygenation (lower AOU) and weak natural variability (Figs. S1o and S2o). Our oxygen ToD at 500 m shares many similar features with the time of emergence (integrated between 100 and 600 m or along $\sigma _\theta =26.5$ density layer) estimated from CMIP5 models^10,30^, namely early emergence in the eastern tropical Atlantic, parts of the Southern Ocean and the Arctic, whereas later emergence is estimated in most of the Pacific and subtropical South Atlantic. At 1000 m and below, most models project deoxygenation throughout the world ocean, particularly in the Atlantic and the Southern Ocean (Fig. S2p–r). Here, the deoxygenation could be linked to the slowdown of the overturning, which increases the ventilation timescale and thus AOU^13^. The ToD in these regions are projected to occur in the mid $21{{\text {st}}}$ century (Fig. 1p–r). The robust early departure in the interior subpolar North Atlantic has also been shown in an earlier study^12^, and is associated with reduced ventilation in the future warmer ocean.

In much of the Pacific, western Atlantic, and Indian Oceans, the earliest oxygen ToD, i.e., before the mid $21{{\text {st}}}$ century, are projected at the surface (Fig. 2c,g). In the subpolar north and eastern upwelling region of the Atlantic as well as in the Arctic and the Southern Ocean, earliest ToDs occur below 500 m. We note that the ToD only imply that projected deoxygenation will exceed the range of natural variability in oxygen; this does not necessarily imply negative consequences for marine biota. Only when the oxygen decline reaches the hypoxia level (${{\text {O}}}_2 < 50\, {{\text {mmol m}}}^{-3}$) will the marine organisms experience physiological stresses^31^.

### pH

Ocean acidification is a well-known consequence of anthropogenic carbon uptake, leading to an increased hydrogen ion concentration in the seawater and decreased pH level^32^. While warming also contributes to pH reduction, the long-term trends of acidification at surface and in the mixed layer are predominantly driven by increasing dissolved inorganic carbon concentration as a result of growing $\text {pCO}_2$ in seawater, which strongly follows the atmospheric ${{\text {CO}}}_2$ pathways (Fig. S2s,t)^33,34^. Regionally, the Arctic is projected to experience the most rapid acidification in the future (Fig. S2s,t)^35^. By the end of the century, much of the absorbed anthropogenic carbon is expected to have reached 1000 m depth, particularly in the intermediate and deep-water formation regions (North Atlantic and Southern Ocean)^19,36,37^. Consequently, the pH decline in these regions is also projected to be stronger than elsewhere (Fig. S2u,v).

Of the analyzed variables, pH change is detectable earliest, primarily due to its large signal-to-noise ratio. In fact, surface pH decline has been shown to emerge from its background natural variability within a decadal time scale and have already emerged in most of the global ocean^11,38^. The pH change is relatively uniform at 0–100 m (Fig. S2s,t) but spatial patterns depicting watermass ventilation regions are evident in the ToD maps for deeper levels (Fig. 1s–v). There are also slight delays of the surface ToD in the upwelling regions of tropical Pacific, polar Southern Ocean, and the North Pacific. These delays are consistent with the larger background variability and weaker pH decline in these regions (Fig. S1t). The impact of the relatively large background variability in delaying ToD in the Pacific is also evident below the mixed layer (Fig. S1v–x). In the deep Atlantic ($>1500\,\text {m}$) on the other hand, the models indicate a robust early ToD, despite weak acidification signals.

Overall, the earliest ocean acidification signals is present in the surface layer before the year 2030. Interestingly, in the Arctic and subpolar North Atlantic, despite having the largest surface acidification signal, the earliest departure (2020s) occurs at 500 m and $>1000\,\text {m}$, respectively, due to the low background variability at these depths (Figs. 2d,h and S1u–x).

### Departure under mitigated scenario

**Figure 5:**
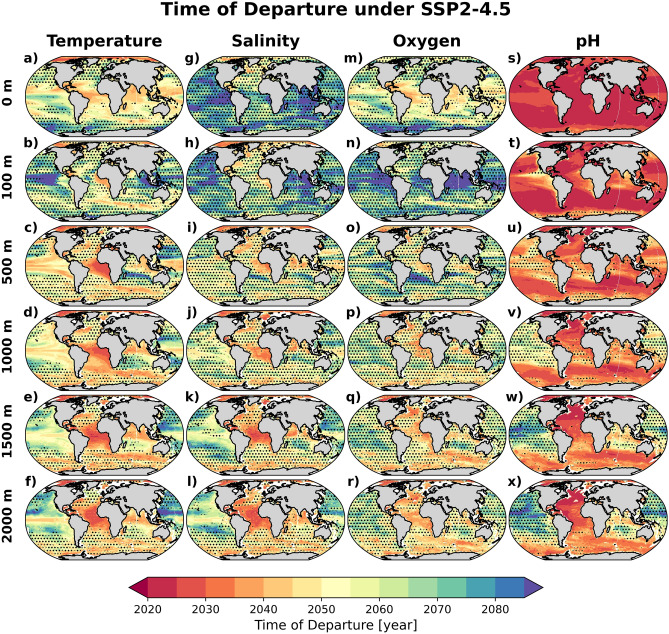
Time of Departure (ToD) maps for SSP2-4.5 scenario. Multi-model mean ToDs for (**a**–**f**) temperature, (**g**–**l**) salinity, (**m**–**r**) oxygen, and (**s**–**x**) pH for surface, 100 m, 500 m, 1000 m, 1500 m and 2000 m depths. Values are estimated from Earth system model projections under the SSP2-4.5 future scenario. The stippling marks areas where the inter-model standard deviation is above 20 years. Maps were generated with Python 3.9.16 using Matplotlib v3.6.2 (https://matplotlib.org/) and Cartopy v0.21.1 (https://scitools.org.uk/cartopy/).

We repeated our analysis with model projections under the SSP2-4.5 scenario to determine the impact of mitigation efforts on the departure time scale. Here, we consider the SSP2-4.5 scenario as the most representative mitigated future scenario based on the ${{\text {CO}}}_2$ emission pledges submitted to the Intended Nationally Determined Contributions^39^. The projected ToDs for the four variables at different depth levels under this scenario are depicted in Fig. 5.

At surface and 100 m levels, emission reductions alleviates anthropogenic climate change signals associated with warming and salinity and oxygen changes, delaying their potential detection timescales by a few decades. Although the acidification rate is also reduced, the pH ToD is not significantly altered. Compared to the SSP5-8.5 scenario (Fig. 1), the spatial ToD patterns under SSP2-4.5 are fairly similar, e.g. early surface warming and deoxygenation are still projected for low latitudes (excluding the tropical Pacific) and the earliest detectable salinity changes occur in the North Atlantic. The similarity in the spatial upper ocean temperature and oxygen ToD patterns (between the two scenarios), with some delays under climate mitigation, is broadly consistent with earlier findings from CMIP5 models^11^.

At 500 m and below, the spatial patterns of the ToD for all four variable are remarkably nearly indistinguishable between the two scenarios. The similarities in the interior ToDs under SSP2-4.5 and SSP5-8.5 can be attributed to two factors. First, the ocean inertia leads to a long response time of the deep ocean to external forcing^40^. The anthropogenically-forced signals that emerge at depth by the end of the century, are the consequence of changes that are already now slowly propagating into the interior. Hence, future mitigation have little impact on the interior changes that would manifest within this century. Secondly, the transfer of anthropogenic signals to the deep ocean is driven by the global overturning circulation that is projected to weaken under the SSP5-8.5 scenario and consequently delay the ToDs. On the other hand, the weakened anthropogenic signal in SSP2-4.5 may be compensated by relatively stronger overturning strength^41,42^, leading to very similar rate of change at depth (see also Fig. S4) and hence ToD as in SSP5-8.5. Interestingly, under the mitigated scenario, delays in the surface changes lead to earlier departure of interior oxygen changes, e.g. in the tropics (Fig. S5c).

An earlier study has indicated that sea surface warming in SSP2-4.5 and SSP5-8.5 diverge considerably only in the second half of this century^3^. This reaffirms that indeed projected interior changes in 2100 are already embedded in a large part of the ocean interior by the mid $21{{\text {st}}}$ century. Hence the projected spatial pattern of the interior temperature changes can be expected to be comparable between the two scenarios. Application of salinity in the subsurface (100 and 500 m) as a proxy for AMOC slow down should still hold for the mitigated scenario given that AMOC slow down prior to 2060 is insensitive to future scenarios^22^. Finally, the similar ToDs between the two scenarios (Fig. S5 as compared to Fig. 2) suggests that future monitoring strategies required to detect interior anthropogenic signals may be scenario independent.

### Departure time scale of compound drivers

**Figure 6:**
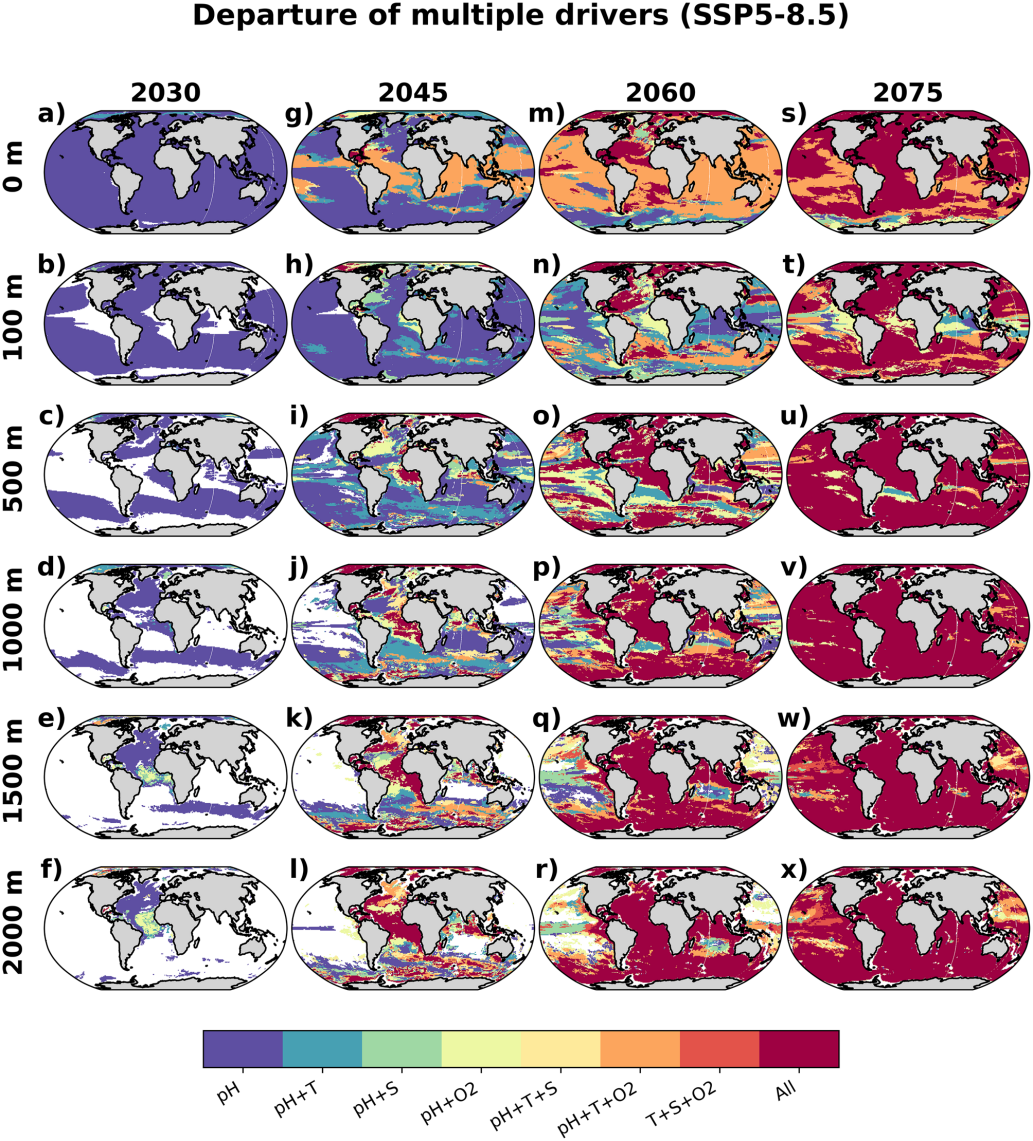
Departure of multiple drivers. Combination of departing variables, based on multi model mean ToD depicted in Fig. 1, in year 2030 (**a**–**f**), 2045 (**g**–**l**), 2060 (**m**–**r**) and 2075 (**s**–**x**), at ocean surface, 100 m, 500 m, 1000 m, 1500 m and 2000 m depths for SSP5-8.5 scenario. Maps were generated with Python 3.9.16 using Matplotlib v3.6.2 (https://matplotlib.org/) and Cartopy v0.21.1 (https://scitools.org.uk/cartopy/).

Figure 6 depicts the departure pattern of multiple drivers at different depths and periods in the $21{{\text {st}}}$ century SSP5-8.5 scenario. In 2030, pH changes are detectable in the entire ocean surface, whilst warming is detectable only in parts of the Arctic. At 100 m, acidification would be detected in most regions, except for the tropical upwelling, the North Pacific, and the polar Southern Ocean (Fig. 6b). Below the mixed layer, intermediate watermasses dominate the pH departure, e.g., Southern Ocean (500–1000 m) and the North Atlantic (500–2000 m). In the deep tropical Atlantic, a combination of either pH+temperature or pH+salinity change would be detected (Fig. 6e,f).

Just prior to the mid 21st century (2045), in addition to the expanding acidification signals, changes in other drivers begin to emerge (Fig. 6g–l). A combination of pH+temperature+oxygen changes emerges in the low latitude surface, except the equatorial Pacific. Below the mixed layer ($>500\,\text {m}$), multi-driver changes are detectable in the Atlantic and the Southern Ocean. The tropical Atlantic emerges as a hotspot, where changes in all four drivers are detectable in the deep ocean already in 2045 (Fig. 6j–l). By 2060, significant changes in at least three drivers are detectable nearly everywhere in the surface ocean. At this time, virtually all drivers would be significantly perturbed by climate change also in the deep Atlantic and the Southern Ocean. The interior Pacific represents the ‘last frontier’ with only a few regions that experience significant changes in all four variables. Finally, toward the end of the century (2075), changes in all drivers are detectable in virtually all parts of the global ocean. A similar pattern is also projected under the mitigation scenario (Fig. S6).

## Observing capacity

The global observing system is well suited to timely detect some of the changes that will unfold in the coming decades. In particular, for temperature, satellites, surface buoys and volunteer observing ships (VOS) ensure ample coverage. At depth, the global ARGO array^43^ in combination with global repeat hydrography^44^ ensure good coverage of both temperature and salinity. The correspondence between AMOC strength and western subtropical Atlantic salinity aligns with evidence from the marine sedimentary record^45^, and is explained by trapping/advection of salt during periods of weak/strong AMOC. While many surface salinity observations are gathered using thermosalinographs on VOS and research vessels^46^, these are not as far as we are aware^47^, regularly compiled into global quality controlled databases, such as done with the surface ocean $\text {pCO}_2$ in SOCAT^48^ for instance. Instigating such an effort seems highly worthwhile given the results presented here.

Important biogeochemical processes such as respiration occur in the mesopelagic zone (200–1000 m). Here, our results indicate that temperature and oxygen properties, which are important to their rates, will change beyond their natural variability within the next few decades. We need to have an observing system in place to detect these property changes and infer their implications on the marine ecosystem and biogeochemistry. In particular, the bulk of organic matter remineralization occurs in the mesopelagic^49^, and is sensitive to temperature^50^. Remineralization is a key aspect of the ocean carbon pump, and its strength in a warming ocean is a key question for the biogeochemical ARGO program. Thus, sustaining this program decades into the future will ensure better understanding of changes in biological-mediated carbon sequestration and also enhance our capacity to detect anthropogenically-forced signals. Our findings indicate that the tropical Atlantic and Pacific, subpolar Southern Ocean and western parts of the subtropical North Atlantic would be suitable areas for long-term regionally focused efforts.

## Summary

We have analyzed the long-term externally forced changes in four marine environmental drivers throughout the water column (0–2000 m) as projected by the latest generation of Earth system models. The goal was to identify ocean regions where anthropogenically-forced signals are robustly projected across the models and have early departure/detectability. Despite the fact that the largest anthropogenic signals have been projected to occur in the upper ocean, the large natural variability could mask these changes from being detected. In contrast, the low background variability in the interior could lead to earlier departure timescale for the gradually propagating anthropogenic signals from surface to depth.

Our analysis indicates that surface warming and deoxygenation patterns are very similar, with detectable changes to occur within the first half of this century in the subtropical oceans. In well ventilated regions of North Atlantic and Southern Ocean, temperature and oxygen changes are projected to be detectable earlier at depth than at surface. The similarity in temperature and oxygen ToD patterns across the models over much of the surface and ventilation regions demonstrates the dominant effect of temperature-driven solubility in these regions. The earliest detectable warming and oxygen changes are projected in the tropical Atlantic at 500 m or deeper. Changes in surface salinity associated with freshwater fluxes are generally detectable only in the second half of this century. On the other hand, salinification of the western subtropical North Atlantic is projected and detectable early, a consequence of a reduction in the Atlantic Meridional Overturning Circulation (AMOC) strength. Therefore, monitoring surface and interior salinity in this domain could provide a robust early warning for a slowdown in AMOC. The model simulations consistently demonstrate early detectable ocean acidification in the upper 100 m, with the exception of the Arctic and subpolar North Atlantic.

Under a mitigated scenario, SSP2-4.5, where atmospheric ${{\text {CO}}}_2$ stabilizes after 2080^51^, the ocean area affected by multiple drivers will continue to expand by 2100, particularly in the deep ocean. This emphasises the slow response time of the ocean interior to change, and further reiterates the need to act early to avoid potentially irreversible degradation of the marine ecosystem from the projected environmental changes identified in this study. Sustained interior observations are crucial to reaffirm the full extent of anthropogenic climate change effects on the world ocean and the subsequent impacts on its ecosystems.

## Methods

We analysed projections from the CMIP6 multi-model ensemble under the high-${{\text {CO}}}_2$ (Shared Socioeconomic Pathway, SSP5-8.5) and mitigation (SSP2-4.5) scenarios for the 2015–2100 period. We downloaded output from 17 Earth system models, which provide 3D annual fields of temperature, salinity, oxygen, and pH (Table 1). To ease processing and comparability, all model outputs were regridded to a common $1^{\circ }\times 1^{\circ }$ grid using a bi-linear interpolation and the specific depth layers (0, 100, 500, 1000, 1500, and 2000 m) were extracted using the nearest neighbour.

**Table 1:** List of the 17 Earth system models from the Coupled Model Intercomparison Project phase 6 (CMIP6), their respective variables (T and S denote temperature and salinity, respectively) and scenarios analyzed in this study, their spin-up durations in model years, horizontal resolutions of the ocean component, and references.

Model name	SSP2-4.5	SSP5-8.5	Spin-up	Resolution	References
ACCESS-ESM1-5	T, S, O2	T, S, O2	1000	$1^\circ \times 1^\circ$	^54^
CanESM5	T, S, O2	T, S, O2	1000	$1^\circ \times 1^\circ$	^55^
CanESM5-CanOE	T, S, O2, pH	T, S, O2, pH	1000	$1^\circ \times 1^\circ$	^55,56^
CESM2	T, S	T, S	1200	$1.125^\circ \times 0.53^\circ$	^57^
CESM2-WACCM	T, S, pH	T, S, pH	500	$1.125^\circ \times 0.53^\circ$	^57,58^
CMCC-ESM2	T, S, O2, pH	T, S, O2, pH	1320	$1^\circ \times 1^\circ$	^59^
CNRM-ESM2.1	T, S, O2, pH	T, S, O2, pH	2600	$0.3^\circ$–$1^\circ$	^60^
EC-Earth3-CC	T, S, O2, pH	T, S, O2, pH	1600	$1^\circ \times 1^\circ$	^61^
GFDL-CM4	T, S, O2, pH	T, S, O2, pH	150	$0.25^\circ \times 0.25^\circ$	^62^
GFDL-ESM4	T, S, O2, pH	T, S, O2, pH	400	$0.5^\circ \times 0.5^\circ$	^63^
IPSL-CM6-LR	T, S, O2, pH	T, S, O2, pH	500	$0.3^\circ$–$1^\circ$	^64^
MPI-ESM1.2-HR	T, S, O2, pH	T, S, O2, pH	2000	$0.4^\circ \times 0.4^\circ$	^65,66^
MPI-ESM1.2-LR	T, S, O2, pH	T, S, O2, pH	12000	$1.5^\circ \times 1.5^\circ$	^66^
MRI-ESM2.0	T, S, O2, pH	T, S	2500	$1^\circ \times (0.3{-}0.5)^\circ$	^67,68^
NorESM2-LM	T, S, O2, pH	T, S, O2, pH	1600	$1^\circ \times 1^\circ$	^69,70^
NorESM2-MM	T, S, O2, pH	T, S, O2, pH	1200	$1^\circ \times 1^\circ$	^69,70^
UKESM1.0-LL	T, S, O2, pH	T, S, O2, pH	500	$1^\circ \times 1^\circ$	^71^

We defined the time of departure (ToD) as the first year when the projected signals emerge outside (above or below) the envelope of natural variability^13^. The following calculations were done for each variable in each individual grid of each model. First, we removed any potential model-specific drift by detrending the values of each variable using the respective preindustrial trend, which was calculated from the last 50-years of each model's preindustrial control simulation. We note that there could be remaining secondary drifts in a few models with relatively short spin-up time (see Table 1). A smoothed time-series for the future scenario projection was then calculated using a 10-year running mean. Next, an average natural variability envelope in each model grid, defined as $\pm 2$ standard deviations ($\sigma$) of the interannual variability from the preindustrial control simulation, was centred around the first value (year 2015) of the smoothed time-series projection. Finally, the ToD was estimated as the first year when the smoothed time-series departs outside the $\pm 2\sigma$ natural variability. An illustration of how the ToD is determined for a single variable in a single model grid is provided in Fig. S7.

We note that the emergence of anthropogenically-forced signals strongly depend on the spatially heterogeneous internal climate variability as well as on intrinsic model variability^4^. Applying a lower or higher threshold for the natural variability envelope (e.g. $\pm 1 \sigma$ or $\pm 3 \sigma$) will lead to earlier or later ToD, respectively. Here, our ToD results are estimated with 2015 as the initial year (i.e., in order to allow clear comparison of ToD and the impact of following different future scenarios). Thus, for variables such as pH, the actual anthropogenically-forced signals are known to have emerged earlier in much of the surface ocean, prior to year 2015^11^. We further note that in our approach, observational uncertainties, e.g., as associated with measurement errors were not considered. In the vertical space, we focused on the region between surface and upper part of the bathypelagic zone (2000 m). This domain comprises the thermocline and has broad biodiversity, for example it is the habitat for the majority of marine fishes^52^. Understanding the time-scale of anthropogenic climate change in this domain is therefore crucial for numerous reasons. From the monitoring perspective, the upper 2000 m also covers the depth range where most ARGO floats collect data.

The ToD is first computed for each individual model. The results presented here represent a multi-model average of ToDs. To illustrate the robustness (or agreement) of ToD values across the different models, we applied stippling overlaying the ToD maps to depict regions where the multi-model standard deviation of ToD is greater than 20 years (indicating large uncertainties). This simple approach highlights regions where the multi-model agreement is relatively low and the respective ToDs are considered to be less robust. We note that it is reasonable to assume that the uncertainty due to multi-model spread under future climate change projection outweighs the uncertainty associated with the spin-up duration and the internal variability simulated in each model^53^. However, we acknowledge that in old watermass regions, such as the interior eastern tropical Pacific, the oxygen ToD could be sensitive to the length of model spin up.

## Supplementary Information

Supplementary Figures.

## Data Availability

Output from 17 IPCC-class Earth system models run for the CMIP6 exercise under piControl, SSP2-4.5 and SSP5-8.5 experiments are publicly available to downloaded from the archive at https://esgf-node.llnl.gov/search/cmip6/. The models and variables analyzed in this study are listed in Table 1.
