# Supplementary material for: Early detection of anthropogenic climate change signals in the ocean interior
Source: Sci Rep. 2023 Feb 21;13:3006. doi: 10.1038/s41598-023-30159-0 (PMC9944908; doi:10.1038/s41598-023-30159-0)
Supplement: Supplementary file 1 — Supplementary Figures. [file 41598_2023_30159_MOESM1_ESM.pdf]

Early detection of anthropogenic climate change signals in the ocean interior

– Supplementary materials –

Jerry F. Tjiputra[1,*], **Jean Negrel**[1], **and Are Olsen**[2]

[1]NORCE Norwegian Research Centre, Bjerknes Centre for Climate Research, Bergen, Norway
[2]University of Bergen, Bjerknes Centre for Climate Research, Bergen, Norway
[*]Corresponding author: jetj@norceresearch.no

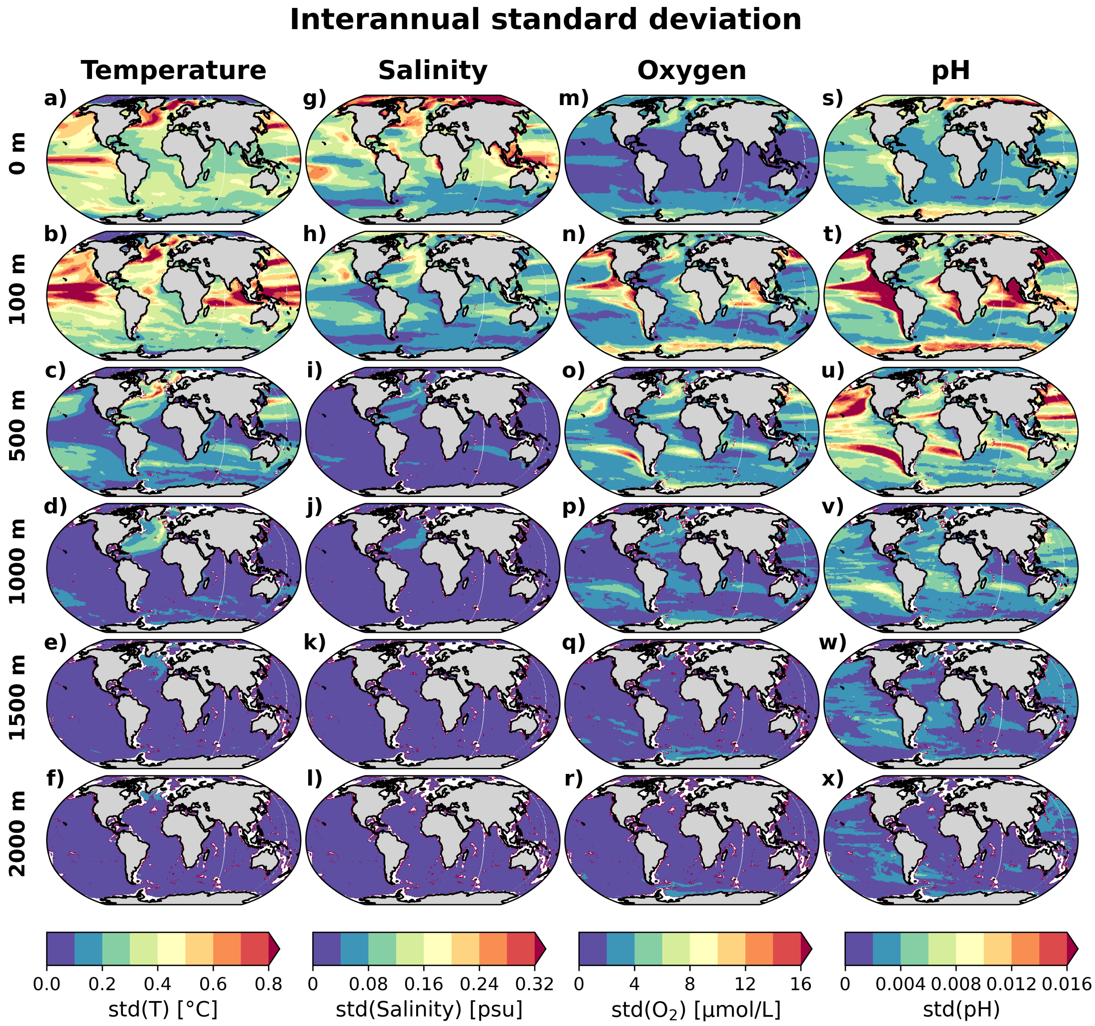

Figure S1. Multi-model average interannual standard deviation of (a-f) temperature, (g-l) salinity, (m-r) oxygen and (s-x) pH at different depths. Maps were generated with Python 3.9.16 using Matplotlib v3.6.2 (https://matplotlib.org/) and Cartopy v0.21.1 (https://scitools.org.uk/cartopy/).

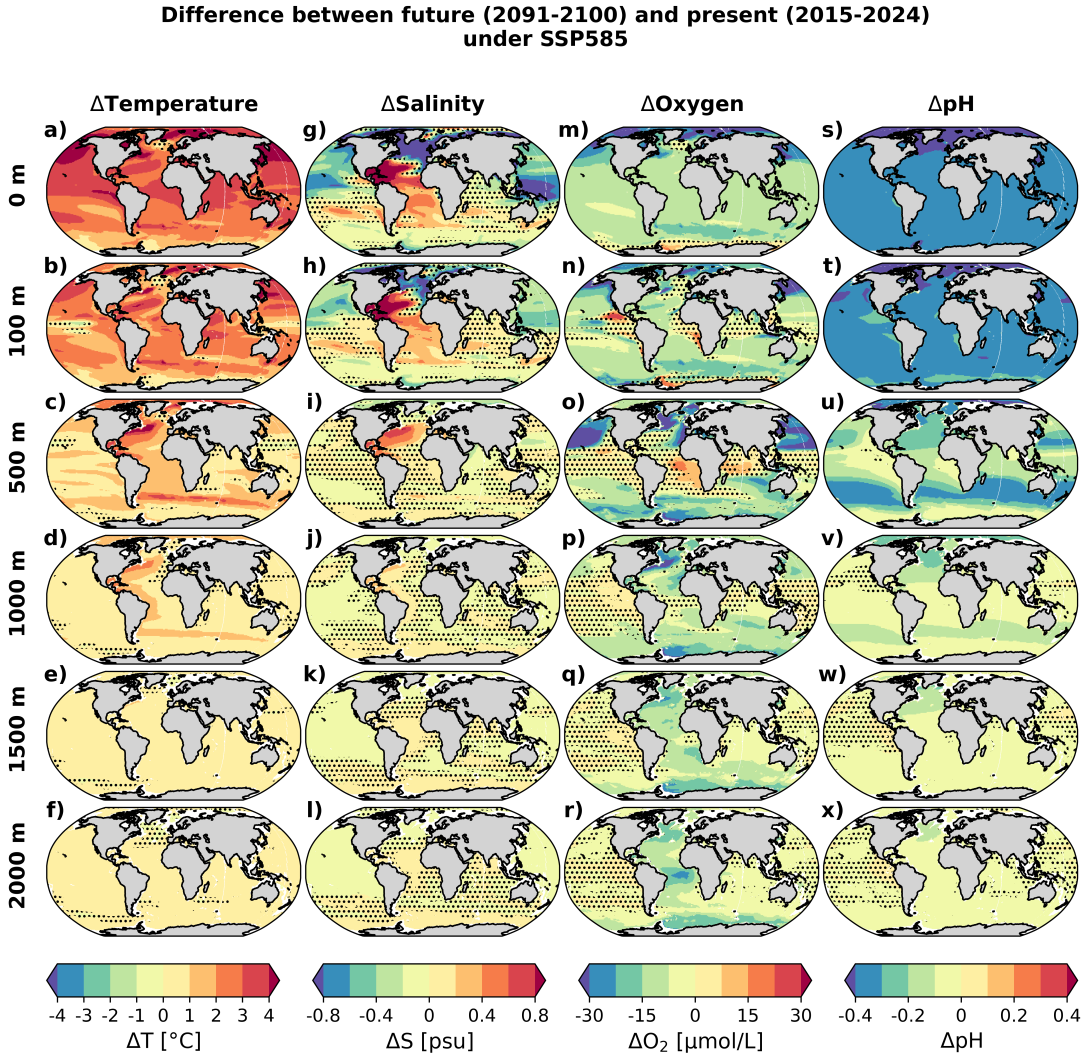

Figure S2. Multi-model average of the difference between future (2091-2100) and present (2015-2024) for (a-f) temperature, (g-l) salinity, (m-r) oxygen and (s-x) pH across the different depth levels under the SSP5-8.5 scenario. Stippling shows areas where less than 75% of the model agree on the direction of the trend. Maps were generated with Python 3.9.16 using Matplotlib v3.6.2 (https://matplotlib.org/) and Cartopy v0.21.1 (https://scitools.org.uk/cartopy/).

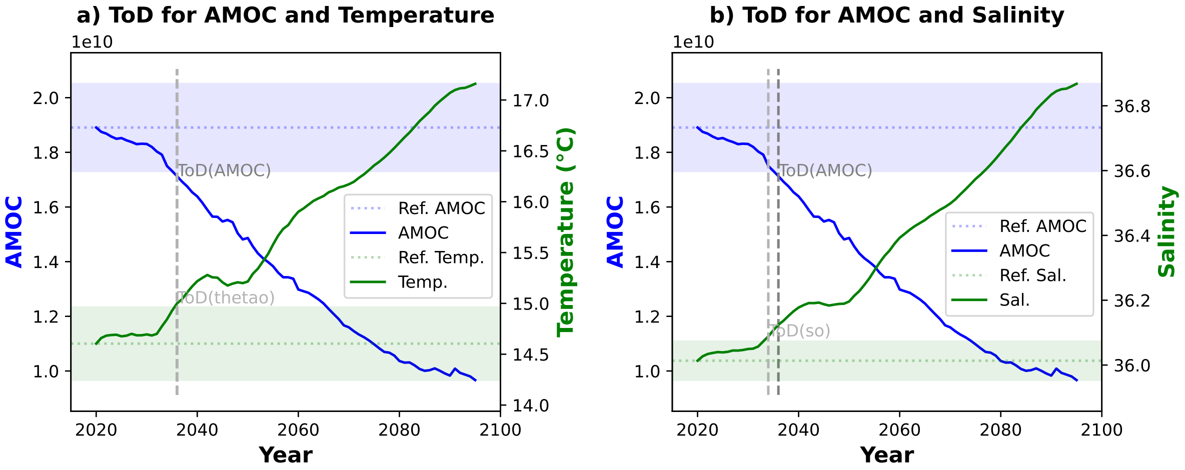

Figure S3. Illustration of the time of departure (ToD) estimation for Atlantic Meridional Overturning Circulation (AMOC) strength [Sv] and temperature (a), and AMOC and salinity (b), at 35°N 55°W and 500 m depth, as simulated by NorESM2-MM. The blue curve represents the 10-year smoothed AMOC strength time-series. The dotted blue line depicts the reference value (50-years average piControl value adjusted to the first value of the blue curve) and the shaded area represents its associated $\pm 2\sigma$. The vertical dashed line is the ToD for AMOC, in this case 2036. The temperature and salinity time-series follow the same plotting convention in green colour. The estimated ToDs for temperature and salinity at this particular point are 2036 and 2034, respectively.

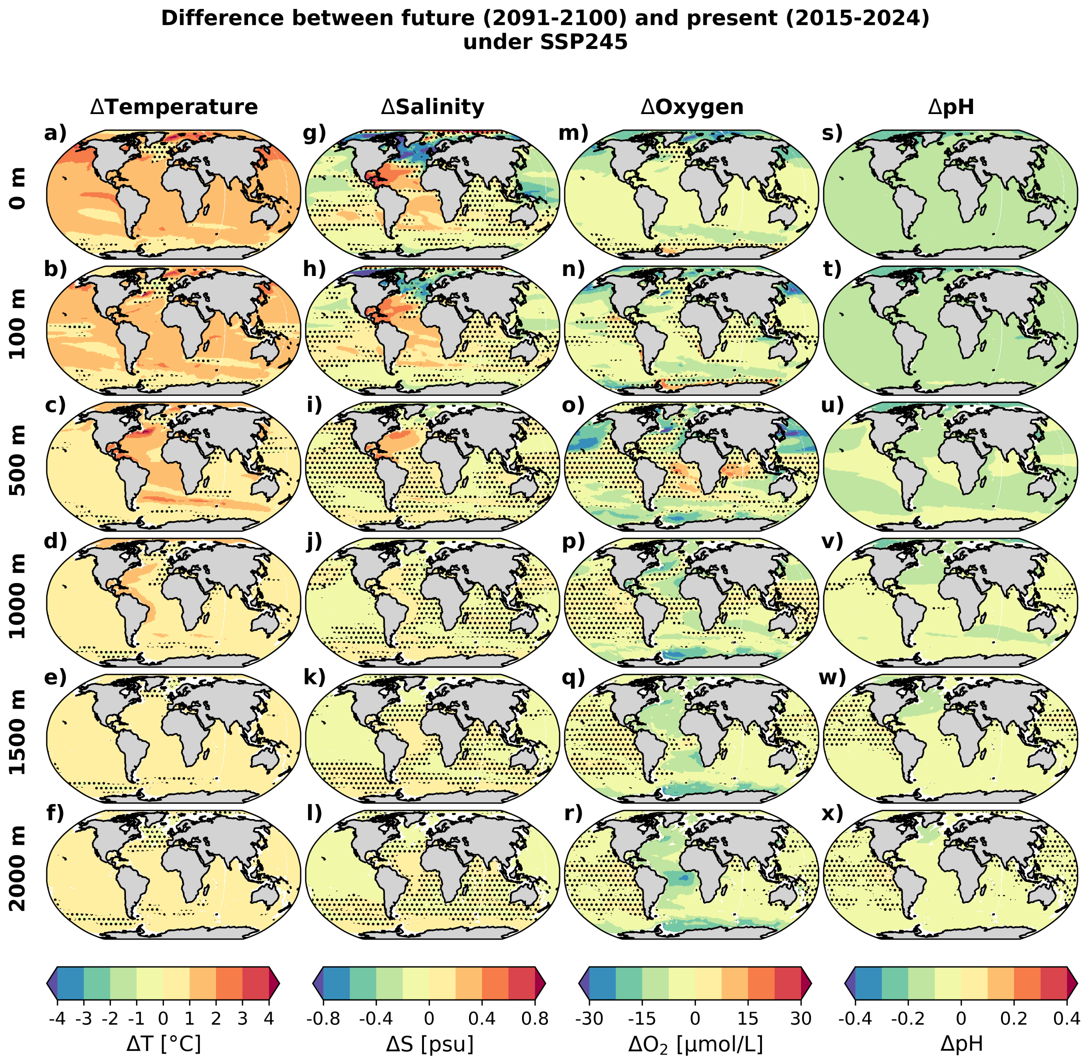

Figure S4. Same as Fig. S2 but for SSP2-4.5 scenario. Maps were generated with Python 3.9.16 using Matplotlib v3.6.2 (https://matplotlib.org/) and Cartopy v0.21.1 (https://scitools.org.uk/cartopy/).

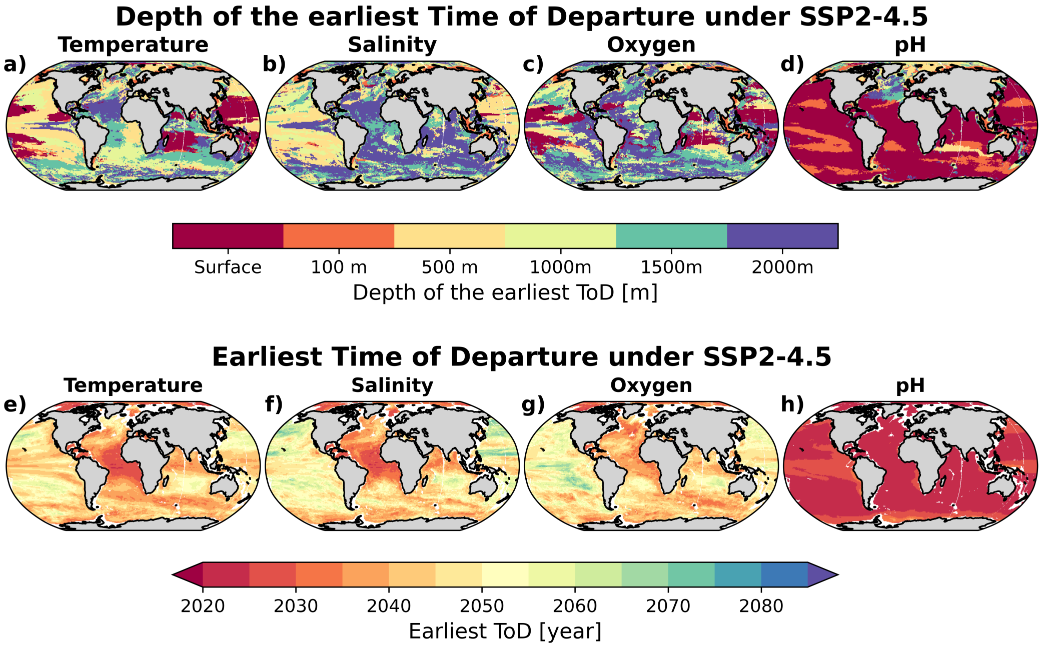

Figure S5. Depth of the earliest departure estimated from the multi-model mean of ToD for (a) temperature, (b) salinity, (c) oxygen, and (d) pH. And earliest departure, considering all depths in panels a-d, in the multi-model mean of ToD for (e) temperature, (f) salinity, (g) oxygen, and (h) pH. Values are estimated from Earth system model projections under the SSP2-4.5 future scenario. Maps were generated with Python 3.9.16 using Matplotlib v3.6.2 (https://matplotlib.org/) and Cartopy v0.21.1 (https://scitools.org.uk/cartopy/).

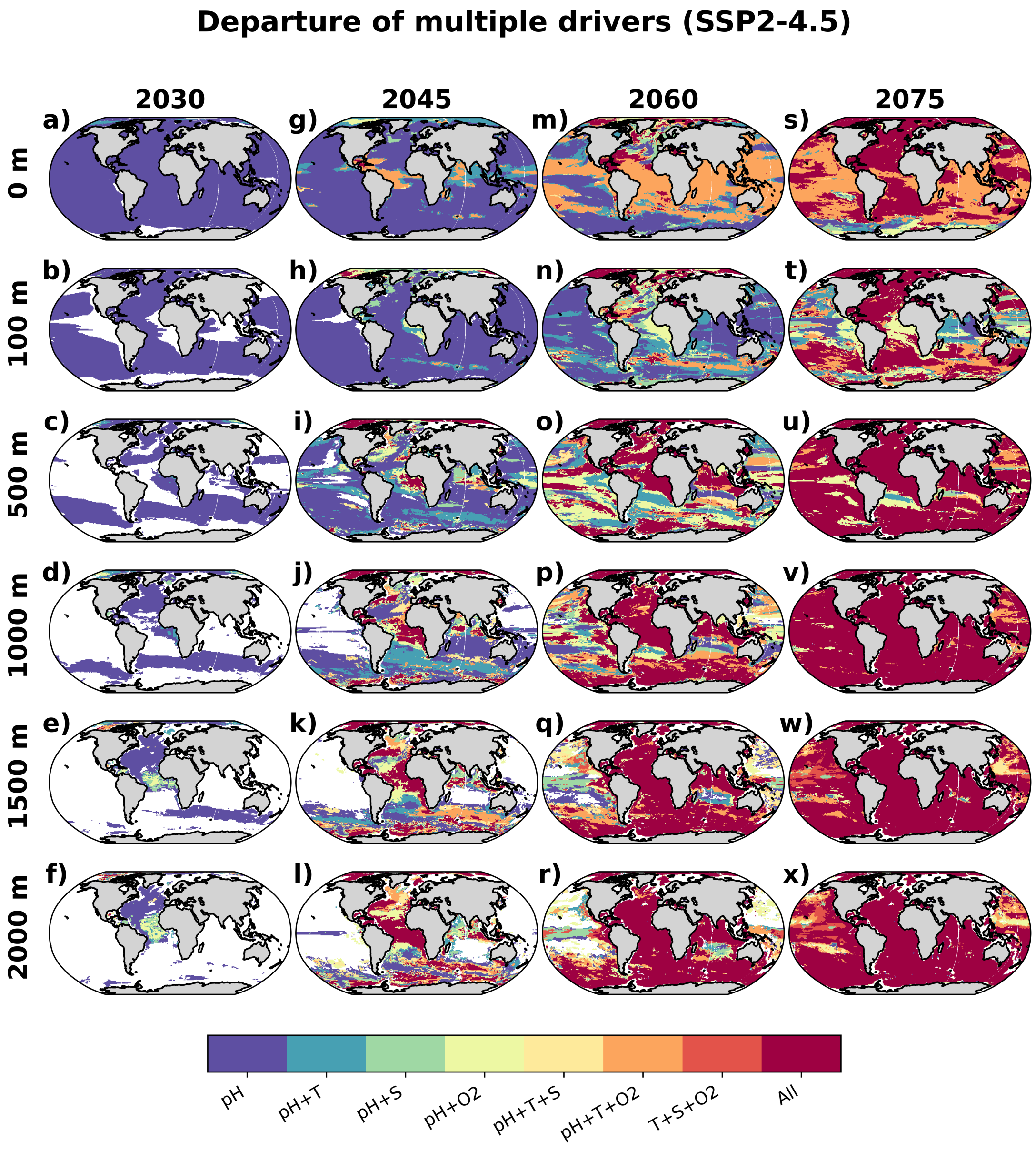

Figure S6. Departure of multiple drivers. Combination of departing variables, based on multi model mean ToD depicted in Fig. 5, in year 2030 (a-f), 2045 (g-l), 2060 (m-r) and 2075 (s-x), at ocean surface, 100 m, 500 m, 1000 m, 1500 m and 2000 m depths for SSP2-4.5 scenario. Maps were generated with Python 3.9.16 using Matplotlib v3.6.2 (https://matplotlib.org/) and Cartopy v0.21.1 (https://scitools.org.uk/cartopy/).

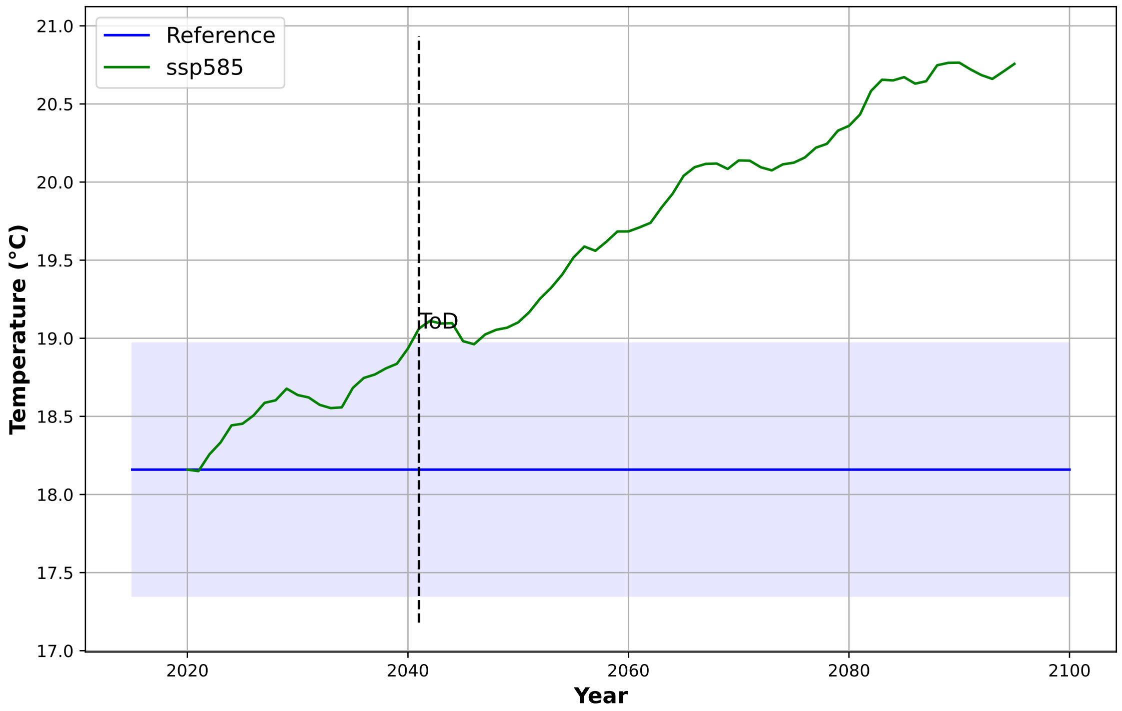

Figure S7. Illustration of the time of departure (ToD) estimation for surface temperature as simulated by GFDL-ESM4 at 34°N 130°W. The green curve represents the 10-year smoothed time-series of the surface temperature at this particular point. The blue line depicts the reference value (50-years average piControl value adjusted to the first value of the green curve) and the shaded area represents its associated $\pm 2\sigma$. The vertical dashed line is the ToD, in this case 2041.
